# Involvement of miRNAs in Placental Alterations Mediated by Oxidative Stress

**DOI:** 10.1155/2014/103068

**Published:** 2014-03-18

**Authors:** Alexander Rudov, Walter Balduini, Silvia Carloni, Serafina Perrone, Giuseppe Buonocore, Maria Cristina Albertini

**Affiliations:** ^1^Department of Biomolecular Sciences, University of Urbino, Via Saffi 2, 61029 Urbino, Italy; ^2^Department of Molecular and Developmental Medicine, University of Siena, Viale Bracci, 36, 53100 Siena, Italy

## Abstract

Oxidative stress (OS) is known to be strongly involved in a large number of fetal, neonatal, and adult diseases, including placental disorders, leading to pregnancy loss and stillbirths. A growing body of research links OS to preeclampsia, gestational diabetes, obesity, spontaneous abortion, recurrent pregnancy, preterm labor, and intrauterine growth restriction. While a considerable number of miRNAs have been related to physiological functions and pathological conditions of the placenta, a direct link among these miRNAs, placental functions, and OS is still lacking. This review summarizes data describing the role of miRNAs in placental pathophysiological processes and their possible impact on OS damaging responses. As miRNAs can be found in circulation, improving our understanding on their role in the pathogenesis of pregnancy related disorders could have an important impact on the diagnosis and prognosis of these diseases.

## 1. Introduction

Oxidative stress (OS) occurs when the production of free radicals exceeds the capacity of antioxidant defenses. It represents an imbalance between the production of reactive species and the biological system's ability to readily detoxify the reactive intermediates or to repair the resulting damage. Each cell is characterized by a particular concentration of electrons stored in many cellular constituents and the redox state of a cell with its oscillation determines cellular functioning [1]. Disturbances in the normal redox state of tissues can cause toxic effects through the production of peroxides and free radicals (FRs) that damage all components of the cell, including proteins, lipids, RNA, and DNA. Some reactive oxygen and nitrogen species (ROS and RNS) can even act as messengers: at low levels, they are signaling molecules and at high levels, they can damage organelles, particularly mitochondria.

Oxidative damage and associated mitochondrial dysfunction may result in energy depletion, accumulation of cytotoxic mediators, and cell death. OS is known to be associated with numerous diseases in humans, such as atherosclerosis, inflammation, cancer, type-2 diabetes, Parkinson's disease, and Alzheimer's disease [2, 3], as well as in various pregnancy related disorders and placenta related alterations [4], such as preeclampsia and intra-uterine growth restriction. However, most mechanisms involved in OS-related pathophysiology in placenta alterations remain unknown. For obvious reasons, pregnancy makes scientific investigation complicated, and most of the knowledge we have on the role of OS during pregnancy is derived from animal studies.

MicroRNAs (miRNAs) are a group of small noncoding RNAs that, after a process of maturation, consist of about 18–25 nucleotides. MiRNAs are known to perform a unique role in posttranscriptional gene regulation. Since their discovery, miRNAs have been known to regulate the expression of a large number of proteins and it is supposed that they could regulate up to 30% (or even more) of the human genome [5]. Indeed, depending on the degree of complementarity, miRNAs can block protein synthesis or even induce mRNA degradation [5]. In addition, members of a microRNA family are clustered and exhibit overlying roles since they share the same sequence for the binding to the mRNA target [6]. At present, the literature describes the importance of miRNAs in the regulation of physiological as well as pathological, cellular, and tissue processes [7, 8]. MiRNAs have been shown to modulate most biological processes, even cellular responses to redox imbalance. In particular, miR-200 family members play a crucial role in OS dependent endothelial dysfunction, as well as in vascular complications of diabetes and obesity [6]. In addition, different miRNAs, such as miR-210, have been shown to play a key role in mitochondrial metabolism, therefore modulating ROS production and sensitivity [9].

MiRNAs have also promising applications in the diagnosis and prognosis of many pathologies, as their quali-quantitative expression varies depending on disease condition [10]. Furthermore, a number of miRNA have already been described as important for the normal placental development and for placenta related alterations [11, 12].

This review will focus on the miRNAs related to placental alterations caused by OS.

## 2. Placental Alterations and Oxidative Stress 

The placenta is a vital organ forming a connection between mother and fetus. It mediates nutrient, oxygen, and hormone exchanges between mother and fetus and operates as an immune protective barrier. Placentation starts with trophoblastic invasion of the maternal spiral arteries and changes in the placental vasculature to ensure optimal maternal vascular perfusion [13]. To ensure a normal fetus growth, equilibrium among trophoblast proliferation, invasion, migration, fusion, and apoptosis is required [14]. However, the regulation and progression of these cellular events remain largely unknown [14]. Several differentiation processes are known to be oxygen regulated via the expression of hormones or growth factors (e.g., the induction of the vascular endothelial growth factor (VEGF) which is triggered via the hypoxia-induced factor) [14]. Furthermore, physiological hypoxia is observable prior to unplugging the maternal spiral arteries by trophoblastic plugs due to the low O_2_ tension in early pregnancy [14]. Unplugging the maternal spiral arteries is followed by an increase of the O_2_ tension with increased ROS production and placental OS [15]. The increase of ROS production maintains important physiological roles and it induces the transcription of several genes related to cell differentiation and proliferation (e.g.,* HIF1A, CREB1, and NFKB1*) [16] and mediates cytokine-induced trophoblast apoptosis [14]. ROS generation leads consequently to a protective up-regulation of antioxidant gene expression and activity of heme oxygenases (HO-1 and HO-2), Cu,Zn-superoxide dismutase (Cu,Zn-SOD), catalase, and glutathione peroxidase (GPx) [17]. However, the high metabolic demands and elevated requirements for tissue oxygen may lead to overproduction of ROS and OS even in normal pregnancies. The loss of the balance of ROS production and antioxidant systems may in fact contribute to a number of serious complications and diseases.

- *Preeclampsia *is a multisystem disorder, that affects 5–14% of pregnancies and is a leading cause of maternal and fetal morbidity and mortality worldwide [18–20]. It manifests itself with focal vasospasm and a porous vascular tree that transfers fluid from the intravascular to the extravascular space causing OS [21]. This state seems to originate from insufficient placental perfusion and a limited enzymatic antioxidant capacity through the reduction of placental Cu,Zn-SOD, GPx and glucose 6-phosphate-dehydrogenase activity and reduced vitamin E tissue levels [22].
- Mechanisms that induce OS seem to be one of the causes of* spontaneous abortion*. Actually, intraplacental circulation is formed 2–4 weeks earlier than under normal conditions [23]. Under such circumstances, the antioxidant enzymes seem not to be present in a sufficient concentration to withstand the OS induced by ROS. This theory is also supported by the presence of increased levels of lipid peroxidation markers and reduced levels of GSH and vitamin E [24].
- *Recurrent pregnancy loss *is a condition that occurs with an incidence of around 3%. A number of studies support the role of OS in the pathophysiology of recurrent pregnancy loss through the increased presence of endometrial NK cells that cause precocious angiogenesis and intraplacental circulation, with a resulting increase in OS [25, 26].
- *Intrauterine growth restriction (IUGR) *refers to a birth weight below the 10th percentile. An important cause of IUGR seems to be preeclampsia, through ischemic mechanisms caused in the placenta and following ROS production [27, 28]. Studies also indicate increased markers of lipid peroxidation in pregnancies with IUGR fetuses [29].
- *Preterm labor *is the leading cause of perinatal morbidity and mortality worldwide with an incidence of up to 12%. Its pathogenic mechanisms include changes in the chorioamniotic membranes with following inflammatory responses [30]. Preterm labor is associated with a ROS-induced reduction of antioxidant defenses (e.g., GPx, GSH) leading to OS, tissue injury, and an increased risk of preterm birth [31, 32].
- *Obesity *during pregnancy is known to be associated with hypertension, diabetes, an increased rate of cesarean section, prematurity, stillbirth, and macrosomia [33]. These complications may be related to OS due to the increased production of inflammatory cytokines, which in turn favor ROS and RNS generation [34].
- *Gestational diabetes *is related to insulin resistance and occurs in 0.3–0.5% of pregnant women [35]. It develops during the second half of pregnancy. Patients with gestational diabetes present a higher risk of spontaneous abortions, perinatal death, congenital anomalies, and disturbances of fetal growth. Oxidative stress seems to be related to gestational diabetes and different markers of OS (e.g., malondialdehyde, Cu,Zn-SOD, and GSH) have been described as being altered under this condition [36].

Since OS has an important impact on placental alterations, a better understanding of the regulatory mechanisms triggered by ROS could be relevant to future research on placental alterations and related complications.

## 3. MicroRNAs Related to Placental Development and Pregnancy 

There is increasing evidence of the importance of miRNAs in placental development [37–39]. A large number of miRNAs and miRNA related proteins (e.g., Drosha, Exportin 5, Dicer, Argonaute 2, and DP103) have been identified in human placental tissues [40, 41]. Additionally, a number of* in vitro *studies [42, 43] and clinical investigations [44, 45] have described the important role of miRNAs in placental development and its alterations. Many miRNAs are known to be specifically expressed in the placenta and three miRNA clusters are known to be specifically associated with placental development.

- *The chromosome 14 miRNA cluster (C14MC) *is the largest described miRNA cluster known, comprising 52 miRNAs. It is located at the human 14q32 chromosome and is expressed from the maternally inherited chromosome. Some miRNAs of the C14MC cluster are predominantly expressed in the placenta. Furthermore, the C14MC seems to play an important role in embryonic development, neurogenesis, and RNA metabolism [46].
- *The chromosome 19 microRNA cluster (C19MC) *is primate specific and comprises 46 miRNAs located at the human 19q13.41 chromosome. C19MC is only expressed from the paternally inherited chromosome and is mainly related to the placenta. Therefore, C19MC may be related to human embryonal development [46].
- *The miR-371-3 cluster *consists of hsa-miR-371a-3p, has-miR-371b-3p, hsa-miR-371-5p, hsa-miR-372, hsa-miR-373-3p, and hsa-miR-373-5p. This cluster is adjacent to the C19MC cluster and is known to be expressed in the placenta. Particularly, the miRNAs of this cluster are highly expressed in human embryonic stem cells (ESCs), while their levels decrease during development. The miR-371-3 cluster seems to regulate cell cycle, proliferation, and apoptosis [46].

Recent studies have revealed that a number of miRNAs act on placental development. Trophoblast proliferation can be promoted bymiR-378a-5p [47], miR-376c [48], and miR-141 [49], while miR-155 [43] and miR-675 [50] have been shown to inhibit trophoblast cell proliferation. MiR-29b has been shown to induce [42] and miR-182 to inhibit apoptosis in trophoblast cells [51]. MiR-195 [52], miR-376c [48], and miR-378a-5p [47] have been reported to enhance and miR-210 [53], miR-34a [54, 55], and miR-29b [42, 54] to inhibit trophoblast migration and invasion. MiR-29b [52] and miR-16 [56] have shown an inhibitory effect on angiogenesis through vascular endothelial growth factor (VEGFA) suppression. Furthermore, a number of miRNAs have been described as being specifically altered in a number of placenta related diseases. Recent studies suggest that miRNAs are involved in the development of preeclampsia [37, 39, 43, 57]. Most notably miR-210 [51, 53], miR-20a [57], miR-20b [57], miR-29b [42], miR-16 [44], miR-155 [58], and miR-675 [50] have shown to be upregulated and are suggested to inhibit angiogenesis, trophoblast cell proliferation, and migration, while miR-378a-5p [47], miR-376c [48], and miR-195 [52] have been shown to be downregulated and to promote trophoblast cell proliferation, survival, and invasion. A study suggests that miR-518b, miR-1323, miR-516b, miR-515-5p, miR-520 h, miR-519d, and miR-526b are downregulated in IUGR [59]. MiR-132, miR-29a, and miR-222 [60] have been described as being underexpressed in gestational diabetes and are suggested as biomarkers of the disease, as their levels are retrievable from circulation. Finally miR-25, miR-338, miR-101, miR-449, miR-154, miR-135a, miR-142-3p, miR-202*, miR-199a*, and miR-136 [61] have been shown to be upregulated in preterm birth. As pointed out, the regulatory mechanism exerted by miRNAs in placental development and alterations is essential but still needs to be more investigated. This includes trophoblast proliferation, differentiation, migration, invasion, and apoptosis as well as angiogenesis and the expression of antioxidant genes. Since different placental alterations seem to show specific miRNA patterns, it is possible to hypothesize that these patterns could be used as biomarkers for these alterations.

## 4. MicroRNAs Modulated by ROS and Probably Related to Placental Alterations 

**Figure 1:**
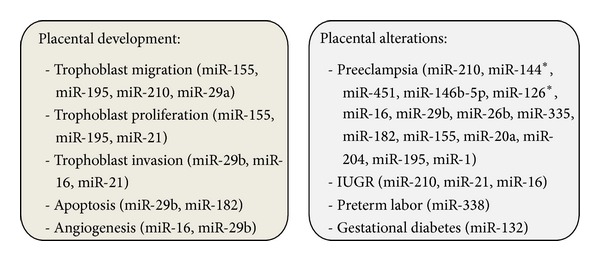
MicroRNAs modulated by oxidative stress. The microRNAs indicated in the figure are involved in placental development/alterations and in pregnancy related disorders.

Some studies suggest that oxygen tension and hypoxia are important regulators of placental miRNA expression [44, 45]. Furthermore, a number of miRNAs previously described as being altered under various placental alterations have been associated with ROS and OS under various experimental conditions. For example, among the miRNAs up-regulated in preeclampsia, miR-210, miR-144*, miR-451, miR-146b-5p, miR-126*, miR-16, miR-29b, miR-26b, miR-335, miR-182, miR-155, and miR-20a [44, 45, 51, 58, 62–64] have been described as being related to OS. MiR-210 was found to be induced by the hypoxia-inducible factor 1-*α* (HIF-1 *α*) [65] and to modulate the adaptive mechanisms involved in acute peripheral ischemia, regulating oxidative metabolism, OS [66], mitochondrial metabolism, angiogenesis, DNA repair, and cell survival [67]. MiR-144 and miR-451 have been found dysregulated under ischaemic conditions in mice and to protect erythrocytes against OS [68]. MiR-144 is known to modulate OS tolerance through down-regulation of the nuclear factor-erythroid 2-related factor 2 (Nrf2), a central regulator of cellular response to OS [69] while miR-451 suppresses the production of 14-3-3zeta (KCIP-1), phosphoserine/threonine-binding protein that inhibits the nuclear accumulation of the transcription factor FoxO3, a positive regulator of erythroid antioxidant genes [70]. MiR-146b-5p has been shown to be downregulated in monocytes of obese subjects and its decrease has been shown to be associated with increased mitochondrial ROS generation and increased NF*κ*B p65 DNA binding activity [71]. A study on the effects of resveratrol on hydrogen peroxide CRL-1730 treated cells showed a downregulation of miR-126 [72]. The induction of OS with N-(4-hydroxyphenyl)-retinamide (4HPR) has been shown to increase the expression of miR-16 and miR-26b in ARPE-19 cells [73]. Chronic OS induces significant downregulation of miR-29b in human trabecular meshwork cells resulting in an increased expression of various extracellular matrix genes [74]. MiR-335 has shown to induce premature senescence of young mesangial cells via suppression of SOD2 and ROS increase [75]. MiR-182 has been shown to be a negative regulator of FoxO1, a protein widely known for its role in protecting diverse cells from ROS [76]. MiR-155 and miR-16 have been found to be altered in their expression in AG01522 primary human fibroblasts (incubated for 1 hour in the logarithmic growth with 25 mM H_2_O_2_) [77]. Interestingly, among these miRNAs, miR-210, miR-155, miR-16, and miR-29b are known for their important roles in normal placental development. MiR-210 is known for its inhibiting role in trophoblast migration, iron metabolism, mitochondrial respiration, and steroid metabolism [53, 62, 63, 78]. MiR-155 regulates negatively trophoblast proliferation and migration, miR-16 inhibits trophoblast invasion, proliferation and angiogenesis, and finally miR-29b inhibits trophoblast invasion and angiogenesis and promotes apoptosis [42, 56, 58]. Among the miRNAs downregulated in preeclampsia, miR-204, miR-195, and miR-1 [45] have been described as being related to OS. MiR-204 has been shown to be an important modulator of OS response in human trabecular meshwork cells, leading to increased levels of apoptosis, decreased viability, and increased accumulation of oxidized proteins [79]. MiR-1, known to occur in ischemic myocardium, has been shown to be upregulated by ROS but downregulated by insulin [80]. A recent study, evaluating the effect of curcumin on altered miRNA expression induced with H_2_O_2_ in ARPE-19 cells, showed also upregulation of miR-195 [81], which has been shown to promote trophoblast invasion [52]. Also miR-338, known to be upregulated in preterm labor, has been described to control axonal ROS levels [82]. MiR-132, downregulated in gestational diabetes [60], has been shown to bear neuroprotective functions against OS through the regulation of PTEN, FOXO3a, and P300, which are all key elements of AKT signaling pathway [83]. The downregulation of miR-21 (as well as the downregulation of the previously mentioned miR-16 and the upregulation of miR-210) has been associated with reduced fetal growth. MiR-21, in addition, has been shown to be upregulated by ROS and to be associated with gastric and colon cancer [84, 85]. Interestingly, miR-21 has also been defined as a functional miRNA since it promotes trophoblast proliferation and invasion [86, 87]. It should be noted that miR-21 and miR-132 have also been found in the maternal circulation [60, 88, 89], highlighting the possibility of using miRNA as diagnostic and/or prognostic tools. OS-related miRNAs deregulated in placental alterations are summarized in Figure 1.

## 5. Concluding Remarks 

ROS production and OS seem to play a key role in placental physiological and pathological conditions. Several miRNAs have been reported to be specifically expressed in the placenta, where they could mediate key processes in its development, like trophoblast proliferation, differentiation, migration, invasion, and apoptosis, as well as angiogenesis and expression of antioxidant enzymes. Interestingly, the expression of some of these miRNAs is altered under various placental alterations, like preeclampsia, gestational diabetes, and other pregnancy related disorders. Some of these miRNAs, such as miR-210, miR-155, miR-16, miR-195, miR-21, and miR-29b, are related to ROS and OS. Considering the important role of OS in placental physiological and pathological conditions, the study of miRNA regulatory mechanisms in this context needs to be intensified especially in the light of the fact that some miRNAs could have an important impact on diagnosis and prognosis of placenta related disorders, as they can be found in circulation. Two promising candidates in this context are miR-21 and miR-132.
